# Effect of intra-arrest transport, extracorporeal cardiopulmonary resuscitation and immediate invasive assessment in refractory out-of-hospital cardiac arrest: a long-term follow-up of the Prague OHCA trial

**DOI:** 10.1186/s13054-024-04901-7

**Published:** 2024-04-16

**Authors:** Daniel Rob, Klaudia Farkasovska, Marketa Kreckova, Ondrej Smid, Petra Kavalkova, Jaromir Macoun, Michal Huptych, Petra Havrankova, Juraj Gallo, Jan Pudil, Milan Dusik, Stepan Havranek, Ales Linhart, Jan Belohlavek

**Affiliations:** 1 2nd Department of Medicine, Department of Cardiovascular Medicine, First Faculty of Medicine, Charles University in Prague and General University Hospital in Prague, U Nemocnice 2, 128 00 Prague 2, Czech Republic; 2 Department of Probability and Mathematical Statistics, Faculty of Mathematics and Physics, Charles University in Prague, Prague, Czech Republic; 3 Czech Institute of Informatics, Robotics and Cybernetics (CIIRC), Czech Technical University in Prague, Prague, Czech Republic; 4 Department of Neurology, First Faculty of Medicine, Charles University in Prague and General University Hospital, Prague, Czech Republic

**Keywords:** Out-of-hospital cardiac arrest, Extracorporeal membrane oxygenation, Extracorporeal cardiopulmonary resuscitation, Long-term, Quality of life

## Abstract

**Background:**

Randomized data evaluating the impact of the extracorporeal cardiopulmonary resuscitation (ECPR) approach on long-term clinical outcomes in patients with refractory out-of-hospital cardiac arrest (OHCA) are lacking. The objective of this follow-up study was to assess the long-term clinical outcomes of the ECPR-based versus CCPR approach.

**Methods:**

The Prague OHCA trial was a single-center, randomized, open-label trial. Patients with witnessed refractory OHCA of presumed cardiac origin, without return of spontaneous circulation, were randomized during ongoing resuscitation on scene to conventional CPR (CCPR) or an ECPR-based approach (intra-arrest transport, ECPR if ROSC is not achieved prehospital and immediate invasive assessment).

**Results:**

From March 2013 to October 2020, 264 patients were randomized during ongoing resuscitation on scene, and 256 patients were enrolled. Long-term follow-up was performed 5.3 (interquartile range 3.8–7.2) years after initial randomization and was completed in 255 of 256 patients (99.6%). In total, 34/123 (27.6%) patients in the ECPR-based group and 26/132 (19.7%) in the CCPR group were alive (log-rank *P* = 0.01). There were no significant differences between the treatment groups in the neurological outcome, survival after hospital discharge, risk of hospitalization, major cardiovascular events and quality of life. Of long-term survivors, 1/34 (2.9%) in the ECPR-based arm and 1/26 (3.8%) in the CCPR arm had poor neurological outcome (both patients had a cerebral performance category score of 3).

**Conclusions:**

Among patients with refractory OHCA, the ECPR-based approach significantly improved long-term survival. There were no differences in the neurological outcome, major cardiovascular events and quality of life between the groups, but the trial was possibly underpowered to detect a clinically relevant difference in these outcomes.

*Trial registration* ClinicalTrials.gov Identifier: NCT01511666, Registered 19 January 2012.

**Supplementary Information:**

The online version contains supplementary material available at 10.1186/s13054-024-04901-7.

## Background

Out-of-hospital cardiac arrest (OHCA) is a leading cause of death in Western countries. Despite extensive efforts to improve OHCA outcomes, the survival rate of hospital discharge remains low, averaging approximately 8% [1]. Most resuscitated OHCA patients do not respond to conventional cardiopulmonary resuscitation (CCPR) and fail to achieve a return of spontaneous circulation (ROSC) [2, 3]. In this context the use of veno-arterial extracorporeal membrane oxygenation (VA ECMO) during ongoing resuscitation, a technique known as extracorporeal cardiopulmonary resuscitation (ECPR), could be a promising intervention in selected patients with refractory OHCA [2–5].

Two single-center, randomized trials (ARREST and Prague OHCA) have presented results suggesting the survival benefit of advanced logistics and ECPR over CCPR at 30 and 180 days [2, 3, 6, 7]. However, in the Prague OHCA trial, ECPR-based approach did not significantly improve survival with neurologically favorable outcome at 180 days compared with CCPR and the trial was possibly underpowered to detect a clinically relevant difference for this outcome [2]. A multicenter, randomized trial (INCEPTION) showed no survival difference between ECPR and CCPR approaches for refractory OHCA at 30 and 180 days post-cardiac arrest [8]. These divergent findings may be attributed to several factors, including variations in system organization, the presence or absence of standardized protocols, different intervals from cardiac arrest to ECPR, case volume and post-resuscitation care. Most importantly, they stress the need for further research as ECPR is resource-intensive, posing significant challenges for prehospital and hospital systems.

Evidence from observational retrospective studies suggests good long-term survival and encouraging but impaired quality of life (QoL) in ECPR patients [5, 9, 10]. However, no randomized data are available on long-term clinical outcomes of the ECPR-based approach in patients with refractory OHCA.

Therefore, we conducted a long-term follow-up of the Prague OHCA trial to assess differences in clinical outcomes between the ECPR-based approach and CCPR and to analyze QoL in long-term survivors.

## Methods

### Study design

The Prague OHCA study was a single-center, prospective, open-label, randomized clinical trial that compared an ECPR-based approach (including early intra-arrest transport, ECPR if ROSC is not achieved prehospital and immediate invasive assessment and therapy) to a CCPR in patients with refractory OHCA: the trial design and results of up to 180 days after OHCA have been published previously [2, 7, 11]. The long-term follow-up of patients was planned and prospectively conducted, but there was no prespecified follow-up statistical analysis plan in the original study protocol and present study is a secondary analysis of RCT [11].

The study was approved by the Institutional Review Board of the General University Hospital and First Faculty of Medicine, Charles University, Prague (192/11 S-IV). Each participant's legal representative was informed of the study enrollment and asked for written informed consent as soon as possible. All patients who regained normal neurological function were asked to provide written permission to use their data. Consent requirements were waived for patients who died at the scene and never reached the hospital and those without known legal representatives. Additional ethical approval was obtained for the long-term follow-up (100/21 S-IV). The trial complied with the Declaration of Helsinki and is registered at www.clinicaltrials.gov (ClinicalTrials.gov Identifier: NCT01511666).

### Participants

Adults aged 18–65 years receiving ongoing resuscitation for witnessed OHCA of presumed cardiac etiology were eligible for enrollment in the trial, given that they had received a minimum of 5 min of advanced cardiac life support without ROSC and when the ECPR team was available at the cardiac center. Patients who had unwitnessed cardiac arrest or presumed noncardiac cause, had suspected or confirmed pregnancy, attained ROSC within 5 min during initial resuscitation, regained consciousness, had obvious lifelimiting comorbidities, bleeding diathesis, known do-not resuscitate order, or known prearrest cerebral performance category (CPC) 3 or greater were excluded [2, 11, 12].

### Randomization and masking

Between March 1, 2013, and October 25, 2020, 264 patients were randomized and 256 enrolled in the study to the ECPR-based arm or CCPR arm using a web-based secured randomization system that assigned patient numbers and intervention groups before hospitalization during ongoing CPR in the field [2]. Randomization into the standard strategy or invasive strategy group was based on 4 strata (men ≤ 45 years, men > 45 years, women ≤ 45 years, women > 45 years), with block size of 8. The block size was not disclosed to research personnel [2]. Functional assessments during follow-up were conducted by qualified evaluators who were blinded to group allocation.

### Long-term follow-up

The follow-up of the present study includes all participants of the original study and initiates at the start of the index event (cardiac arrest) for all patients. All survivors of the index hospitalization were invited to the planned follow-up, including routine outpatient visits to the Heart Failure Center of the General University Hospital in Prague. The schedule for all visits at the outpatient clinic was initially set for 180 days after the index event and continued every six months thereafter. Additional visits were arranged as necessary, based on the patient's clinical status. All-cause mortality and events were determined based on follow-up data and hospital records and confirmed by mortality data from the Czech Central Insurance Database. Any clinical event was verified by hospital or general practitioner records. Long-term follow-up was performed by a cardiologist, a neurologist and a study nurse, either in the outpatient clinic or by telephone. Neurologic outcome during the follow-up was assessed by a neurologist masked to treatment allocation using CPC scores [12]. The CPC scale ranges from 1 to 5, with 1 representing good cerebral performance or minor disability, 2 moderate disability, 3 severe disability, 4 coma or vegetative state and 5 brain death. In addition, a structured interview was done with a functional status questionnaire (the EQ5D5L, www.euroqol.org) and a modified Rankin scale (mRS) with a study nurse masked to treatment allocation. The modified Rankin scale ranges from 0 to 6, with 0 indicating no symptoms, 1 no clinically significant disability, 2 slight disability, 3 moderate disability, 4 moderately severe disability, 5 severe disability and 6 death [13].

### Intention-to-treat, crossovers, as-treated, per protocol population

During the trial, crossovers from the CCPR strategy to the ECPR-based strategy (and vice versa) occurred [2]. In the CCPR to ECPR-based strategy, the decision was made based on the request of an emergency physician. At least two additional unsuccessful defibrillations were required after randomization before the cardiac center coordinator accepted a crossover. The crossover from ECPR to the CCPR strategy was accepted when continuing care with invasive measures was deemed futile. All crossovers occurred during the initial CPR phase (no late crossovers transpired).

The primary analysis of the current study endpoints is done according to the randomization group, and data from patients who crossed over were analyzed by the original group assignment respecting the intention-to-treat principle. The as-treated analysis is a post hoc analysis that pooled all randomized patients according to their treatment allocation after the accepted crossover. The per-protocol analysis is a post hoc analysis that includes only those patients who completed the treatment originally allocated (excluding all crossovers). Some 20/256 patients (7.8%) were crossed over (11 crossovers from the CCPR group to the ECPR-based group and 9 from the ECPR-based group to the CCPR group). Details about crossovers appear in the original report [2].

### Study endpoints

The primary outcome of the long-term follow-up was survival. The secondary outcome was neurological outcome assessed by CPC [12] (a CPC of 1–2 was considered a good neurological outcome and a CPC of 3–5 a poor neurological outcome) and mRS scores (a mRS score of 0–3 was regarded as a good outcome and a score of 4–6 a poor outcome) [13]. Further exploratory outcomes included the occurrence of major events after discharge (all-cause death, all-cause hospitalization, all-cause cardiovascular hospitalization, myocardial infarction, stroke, hospitalization for heart failure and ventricular arrhythmias), assessment of symptoms of heart failure using the New York Heart Association (NYHA) classification and QoL using the EQ5D5L (www.euroqol.org) questionnaire and the EQ visual analog scale.

### Statistical analysis

Data were analyzed according to the intention-to-treat principle, with an additional analysis according to the as-treated and per-protocol principle for the primary and secondary outcome. All exploratory outcomes were analyzed according to the intention-to-treat principle only. Sample size determination of the original study was computed for the 180-day outcomes [11]; there was no formal power analysis for the long-term follow-up. Differences in survival rates were assessed using the Kaplan–Meier estimator with the log-rank test. To compare the treatment arms other endpoints were evaluated by the χ2test or the exact Barnard method for binary endpoints. Differences in EQ visual analogue scale were assessed using Welch's t-test. For the construction of 95% confidence intervals and corresponding p-values of rehospitalizations, the test of relative risk was employed. A two-sided *P* < 0.05 was considered statistically significant. Statistical analyses were performed using the R (R Core Team, 2021) software, version 4.2.3 [14].

## Results

### Patients and follow-up

**Fig. 1:**
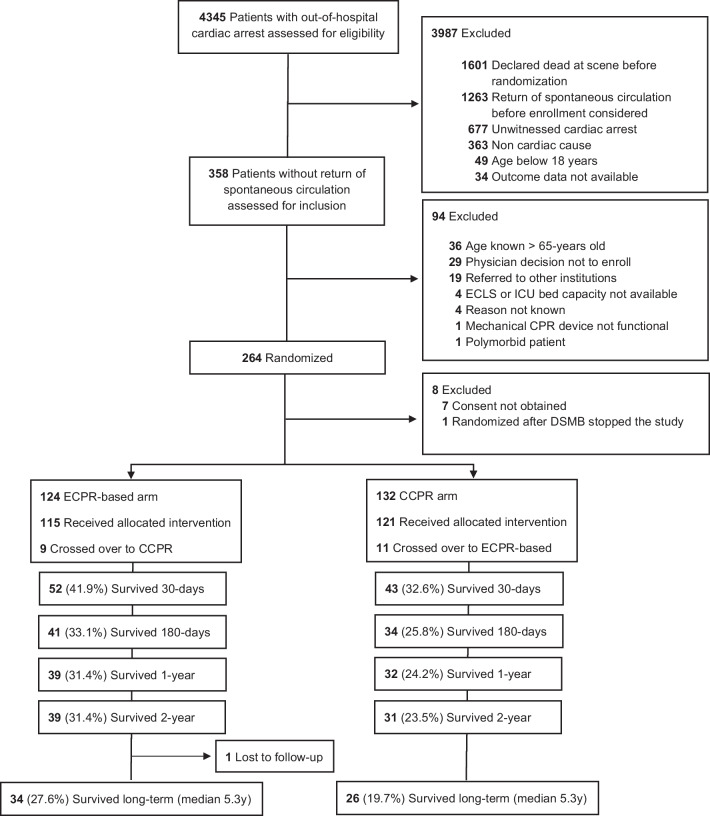
Trial profile. *CCPR* conventional cardiopulmonary resuscitation, *CPR* cardiopulmonary resuscitation, *ECPR* extracorporeal cardiopulmonary resuscitation

From March 2013 to October 2020, 264 patients were randomized during ongoing resuscitation on scene, and 256 (97%) were eligible for the final analysis. Data on screening, randomization, crossovers, 30-day, 180-day, 1-year, 2-year and long-term survival are displayed in Fig. 1. The median long-term follow-up was 5.3 years (interquartile range, IQR 3.8–7.2 years) after initial randomization and was completed in 255 of 256 patients (99.6%). The last follow-up visit was performed on 23 May 2023, > 10 years after the randomization of the first patient on 12 May 2013.

### Baseline and cardiac arrest characteristics

Baseline characteristics, published previously [2], were well balanced between treatment groups. The median age at randomization was 59 years (IQR 48–66) in the ECPR-based group and 57 years (IQR 47–65) in the CCPR group; 82% of patients in the ECPR-based and 83% in the CCPR group were men. Ventricular fibrillation was the most common initial rhythm (72/124 patients (58%) in the ECPR group and 84/132 (64%) in the CCPR group) [2]. Patients were randomized during ongoing CPR after a median of 24 min (min) (IQR 21–30) in the ECPR-based group and 26 min (IQR 19–31) in the CCPR strategy group after the collapse [2].

### Long-term survival

**Fig. 2:**
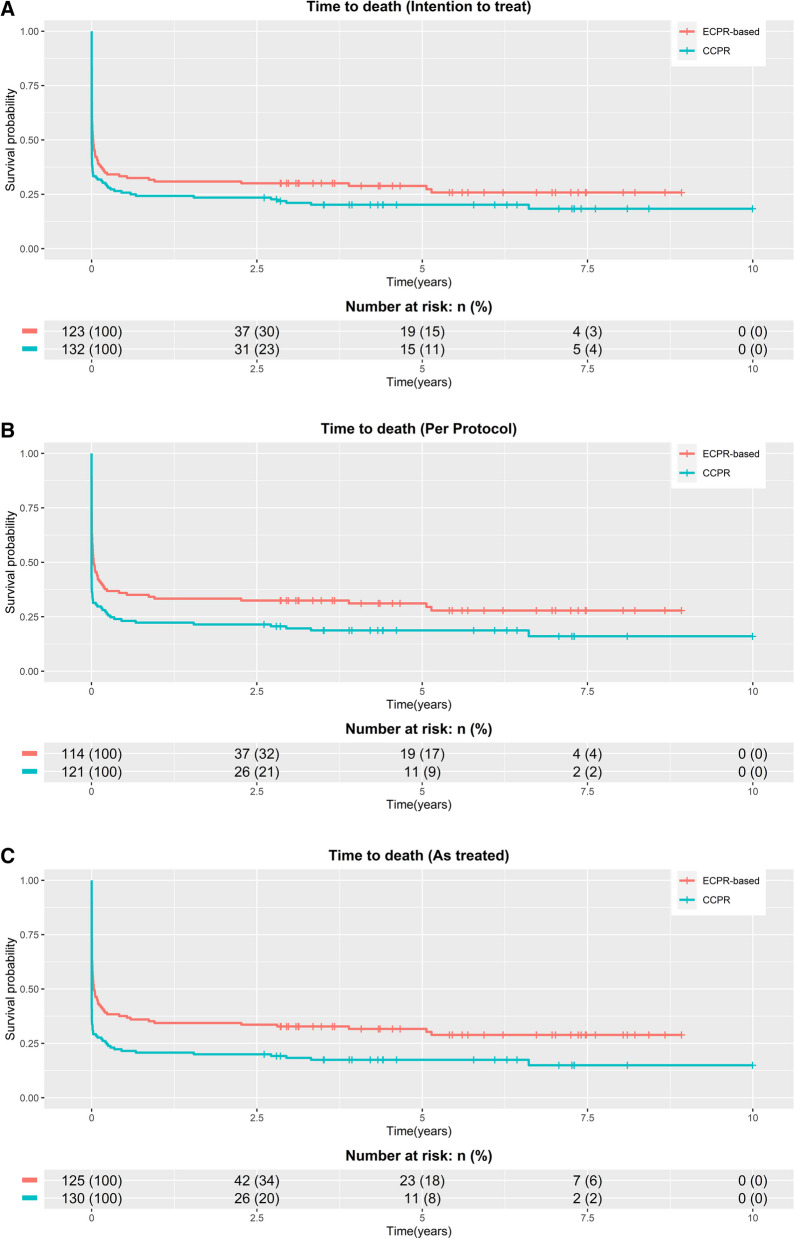
**A** Kaplan–Meier plot showing cumulative patient survival from index cardiac arrest to last follow-up for the intention-to-treat population. *CCPR* conventional cardiopulmonary resuscitation, *ECPR* extracorporeal cardiopulmonary resuscitation. **B** Kaplan–Meier plot showing cumulative patient survival from index cardiac arrest to last follow-up for the per-protocol population. *The per-protocol analysis is a post hoc analysis that includes only those patients who completed the treatment originally allocated (excluding all crossovers, 20/256 patients (7.8%) were crossed over, 11 crossovers from the CCPR group to the ECPR-based group and 9 from the ECPR-based group to the CCPR group). *CCPR* conventional cardiopulmonary resuscitation, *ECPR* extracorporeal cardiopulmonary resuscitation. **C** Kaplan–Meier plot showing cumulative patient survival from index cardiac arrest to last follow-up for the as-treated population. *The as-treated analysis is a post hoc analysis that pooled all randomized patients according to their treatment allocation after the accepted crossover (20/256 patients (7.8%) were crossed over, 11 crossovers from the CCPR group to the ECPR-based group and 9 from the ECPR-based group to the CCPR group). *CCPR* conventional cardiopulmonary resuscitation, *ECPR* extracorporeal cardiopulmonary resuscitation

In the intention-to-treat population, 34/123 patients (27.6%) in the ECPR-based group and 26/132 patients (19.7%) in the CCPR group were alive at the last follow-up (log-rank *P* = 0.01) (Fig. 2A).

For the per-protocol population, 34/114 patients (29.8%) in the ECPR-based group and 22/121 patients (18.2%) in the CCPR group were alive at the last follow-up (log-rank *P* = 0.008) (Fig. 2B).

For the as-treated population 38/125 patients (30.4%) in the ECPR-based group and 22/130 patients (16.9%) in the CCPR group were alive at the last follow-up (log-rank *P* < 0.001) (Fig. 2C).

### Long-term neurological outcome

In the intention-to-treat population, no significant differences were observed between the treatment groups in the CPC and mRS categories (Table 1). A good neurological outcome (CPC 1 or 2) occurred in 33/123 patients (26.8%) in the ECPR-based group and 25/132 patients (18.9%) in the CCPR group (RR 0.90, CI 0.79–1.03, *P* = 0.13). Similar results were found for the mRS category (Table 1).

**Table 1:** Neurological outcome of patients assessed by CPC and mRS at the last follow-up (median 5.3 years, IQR 3.8–7.2 years), by treatment groups, intention-to-treat analysis

CPC category	ECPR-based (*n* = 123)	CCPR (*n* = 132)	*P* value*
1	30 (24.4%)	25 (18.9%)	0.133
2	3 (2.4%)	0	
3	1 (0.8%)	1 (0.8%)	
4	0	0	
5	89 (72.4%)	106 (80.3%)	
mRS category			P value*
0	2 (1.6%)	6 (4.5%)	0.133
1	17 (13.8%)	12 (9.1%)	
2	12 (9.8%)	7 (5.3%)	
3	2 (1.6%)	0	
4	1 (0.8%)	0	
5	0	1 (0.8%)	
6	89 (72.4%)	106 (80.3%)	

*CCPR* conventional cardiopulmonary resuscitation, *CPC* cerebral performance category, *ECPR* extracorporeal cardiopulmonary resuscitation, *mRS* modified Rankin scale

*The *P*-value testing was conducted for CPC 1–2 versus CPC 3–5 and mRS 0–3 versus 4–6

**Fig. 3:**
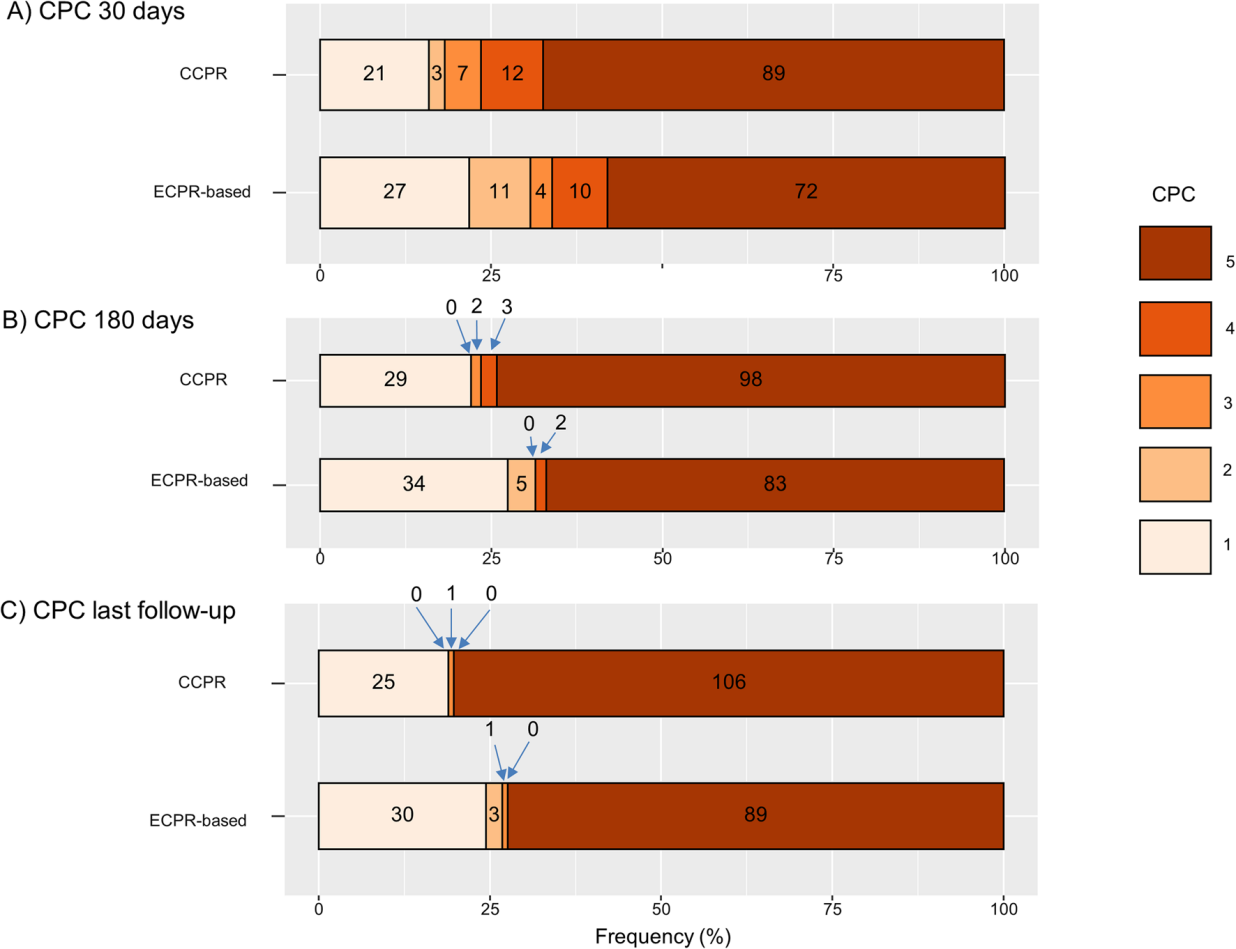
Neurological outcome results assessed by CPC at 30-day (**A**), 180-day (**B**) and the last-follow-up (median 5.3 years, IQR 3.8–7.2 years) (**C**) for CCPR group and ECPR-based group. *CCPR* conventional cardiopulmonary resuscitation, *CPC* cerebral performance category, *ECPR* extracorporeal cardiopulmonary resuscitation

Among long-term survivors, only 1/34 patients (2.9%) in the ECPR-based group and 1/26 patients (3.8%) in the CCPR group had a poor neurological outcome (both with CPC scores = 3) (Table 1). The evolution of neurological outcome results assessed by CPC between 30-day, 180-day, and the last follow-up for ECPR-based and CCPR group is depicted in Fig. 3A–C. The numbers of patients in each CPC category at 30-day, 180-days, 1-year, 2-year, and the last follow-up are described in Additional file 1: Table S1.

For the per-protocol population, a good neurological outcome (CPC 1 or 2) occurred in 33/114 patients (28.9%) in the ECPR-based group and 21/121 patients (17.4%) in the CCPR group (RR 0.86, CI 0.75–0.99, *P* = 0.035). Similar findings were observed for the mRS category (Additional file 1: Table S2).

In the as-treated population, a good neurological outcome (CPC 1 or 2) occurred in 37/125 patients (29.6%) in the ECPR-based group and 21/130 patients (16.2%) in the CCPR group (RR 0.84, CI 0.73–0.96, *P* = 0.007). Similar findings were observed for the mRS category (Additional file 1: Table S3).

### Long-term risk of events and rehospitalization

During the follow-up, 39/123 patients (31.7%) in the ECPR-based group and 30/132 (22.7%) in the CCPR group were discharged from the hospital or long-term hospital facilities after the index event (*P* = 0.11) (median time to discharge 19.5 days, IQR 12.5–32 days). Of these, 4/39 (10.3%) patients in the ECPR-based group and 6/30 (20%) in the CCPR group died during the follow-up (relative risk 0.51 [0.16–1.66], *P* = 0.26). Detailed causes of death are provided in the Additional file 1: Table S4. At least one rehospitalization occurred in 30/39 patients (76.9%) in the ECPR-based group and 18/30 (60%) in the CCPR group (relative risk, RR 1.28 [95%CI 0.91–1.8], *P* = 0.15). At least one cardiovascular rehospitalization occurred in 25/39 patients (64.1%) in the ECPR-based group and 15/30 (50%) in the CCPR group (RR 1.28 [95%CI 0.84–1.97], *P* = 0.26). The frequency of major cardiovascular events, presented in Table 2, was low in both groups. Most of these rehospitalizations were attributable to staged cardiovascular procedures, and details are provided in Additional file 1: Table S5.

**Table 2:** Clinical events after index hospitalization during follow-up among patients discharged home from the hospital or long-term facility, by treatment groups, intention-to-treat analysis

Event	ECPR-based (*n* = 39)	CCPR (*n* = 30)	Relative risk (95% CI)	*P* value
All-cause death	4 (10.3%)	6 (20%)	0.51 [0.16–1.66]	0.26
All-cause hospitalization	30 (76.9%)	18 (60%)	1.28 [0.91–1.8]	0.15
All-cause cardiovascular hospitalizations	25 (64.1%)	15 (50%)	1.28 [0.84–1.97]	0.26
Myocardial infarction	1 (2.6%)	1 (3.3%)	NA	0.91
Stroke	0	1 (3.3%)	NA	0.34
Heart failure hospitalization	2 (5.1%)	3 (10%)	NA	0.57
Hospitalization for ventricular arrhythmia	1 (2.6%)	3 (10%)	NA	0.22

*CCPR* conventional cardiopulmonary resuscitation, *ECPR* extracorporeal cardiopulmonary resuscitation

### Functional status and quality of life

Among long-term survivors, 57/60 (95%) were in NYHA class I or II (94.1% in the ECPR-based group and 96.1% in the CCPR group, *P* = 0.74). Details of the health profile assessed by the EQ-5D-5L are summarized in Table 3. There were no significant differences in QoL between the two treatment groups. The mean EQ-VAS value was 71.0 (± 19.9) in the ECPR-based group and 76.3 (± 18.3) in the CCPR group (*P* = 0.30).

**Table 3:** EQ-5D results, numbers and percentages of patients reporting problems in different dimensions at the last follow-up (median 5.3 years, IQR 3.8–7.2 years), by treatment groups, intention-to-treat analysis

EQ5D dimension	ECPR-based (*n* = 34)	CCPR (*n* = 26)	*P* value*
*Mobility*			
Level 1	21 (61.7%)	17 (65.4%)	
Level 2	6 (17.6%)	3 (11.5%)	1
Level 3	4 (11.8%)	3 (11.5%)	
Level 4	3 (8.8%)	3 (11.5%)	
Level 5	0	0	
*Self-Care*			
Level 1	28 (82.3%)	21 (80.8%)	
Level 2	3 (8.8%)	2 (7.7%)	0.79
Level 3	2 (5.9%)	2 (7.7%)	
Level 4	1 (2.9%)	1 (3.8%)	
Level 5	0	0	
*Usual activity*			
Level 1	25 (73.5%)	21 (80.8%)	
Level 2	3 (8.8%)	0	0.96
Level 3	3 (8.8%)	4 (15.4%)	
Level 4	3 (8.8%)	1 (3.8%)	
Level 5	0	0	
*Pain/discomfort*			
Level 1	18 (52.9%)	17 (65.4%)	
Level 2	10 (29.4%)	7 (26.9%)	0.34
Level 3	4 (11.8%)	2 (7.7%)	
Level 4	2 (5.9%)	0	
Level 5	0	0	
*Anxiety/depression*			
Level 1	24 (70.6%)	22 (84.6%)	
Level 2	7 (20.6%)	2 (7.7%)	0.96
Level 3	3 (8.8%)	2 (7.7%)	
Level 4	0	0	
Level 5	0	0	

*CCPR* conventional cardiopulmonary resuscitation, *ECPR* extracorporeal cardiopulmonary resuscitation

*The *P*-value testing was conducted for Level 1 + 2 versus Level 3 + 4 + 5

## Discussion

In this long-term follow-up to a randomized controlled trial, an ECPR-based approach to refractory OHCA was associated with a significant survival benefit compared to CCPR. The survival benefit was observed in the intention-to-treat, per-protocol and as-treated populations. The importance of this finding is underlined because most patients in this cohort are middle-aged adults (the median age in the ECPR-based group was 59 years) with prolonged resuscitations. Moreover, our data suggest that the ECPR-based approach as a resource-intensive method translates into long-term benefits.

In terms of neurological outcomes, in both the CPC and mRS assessments, our study did not identify a significant difference between the study groups in the intention-to-treat analysis. This finding is consistent with the results observed at the 180-day mark [2]. However, it is important to note that the trial may have been underpowered to detect a clinically relevant difference. In contrast to the intention-to-treat analysis, both the per-protocol and as-treated analyses of neurological outcomes demonstrated a benefit of the ECPR-based strategy over CCPR. Nevertheless, these findings should be interpreted cautiously as they are hypothesis-generating only. A larger RCT to address neurological outcomes associated with the ECPR-based and CCPR strategies, including an assessment of minimal or no neurological impairment, is imperative.

Limited data are available on long-term outcomes in the refractory OHCA population [9, 10]. A retrospective observational analysis of patients who received ECPR for refractory ventricular fibrillation from Minnesota showed a 27% survival at 1 year, close to our results with a 1-year survival of 31% in the ECPR-based group [9]. Another retrospective analysis of the consecutive in-hospital cardiac arrest and OHCA cases treated with ECPR from Germany also showed 31% survival at 1 year [10]. The Minnesota analysis focused on survival and compared OHCA survivors to patients with heart failure who received heart transplantation or a left ventricular assist device and did not provide data on neurological outcome and QoL [9].

Another important finding is comparable survival between the ECPR-based and CCPR groups after discharge home from the hospital or long-term hospital facilities. This finding aligns with data from a Danish retrospective observational study showing similar survival rates between OHCA survivors treated with CCPR or mechanical circulatory support devices after hospital discharge [15]. Findings from our and the Danish study also suggest that overall long-term survival after discharge (90% in our ECPR-based group and 89% in the Danish study) is comparable to that seen in patients with short time to ROSC, who have the highest chance of in-hospital survival.

An additional noteworthy result of this study is that only two long-term survivors exhibited advanced neurological impairment with no difference between the ECPR-based and CCPR groups. However, these patients regained consciousness but remained dependent on long-term care. This result has several important implications. First, it suggests that severe neurological impairment is rare in refractory OHCA survivors after 180 days. Secondly, it underscores the significance of long-term survival as a relevant endpoint for follow-up. Our results are supported by observational studies [10, 15] and a randomized trial [16] reporting good neurological outcomes in most OHCA survivors.

Contrary to these findings from European centers, an observational study from South Korea [17] reported a high proportion of severe neurological impairment in OHCA survivors treated with CCPR at 1 year (34% of patients with a CPC score of 3 or 4). These poor neurological outcomes in the South Korean study were probably caused by low rates (36%) of bystander CPR and an initial shockable rhythm (15%). Our results are derived from a selected refractory OHCA population with high rates of bystander CPR (99%) and initial shockable rhythms (60%) and are therefore not generalizable to OHCA all-comers. Moreover, decisions regarding prognostication and withdrawal of life-sustaining therapy in patients with severe neurological impairment may differ substantially between centers, countries and regions, which may influence these results.

Moreover, few studies with limited sample sizes provided insights into the long-term neurological outcome evolution after OHCA [17–19]. Significant changes in the neurological outcome were observed between 1 and 6 months, with almost no changes occurring after 6 months, except for death. Our study confirms these findings in a randomized refractory OHCA population. However, further research in a larger population is needed as neurological recovery and outcome evolution have important consequences for long-term care decisions and outcome selection in future clinical studies. The commonly used 1-month outcomes in OHCA trials are too short to assess the effects of interventions in this population and a longer primary follow-up is needed [17–19].

Long-term follow-up data on patients discharged home after the index event in our study revealed a high number of rehospitalizations. Although the total number of major adverse cardiovascular events was relatively low, the high number of hospitalizations following discharge deserves attention. We found no study focusing on the risk of cardiovascular events and admission to hospital in OHCA survivors. However, this finding is not surprising, given that many OHCA patients have comorbidities [2, 20] and severe coronary artery disease as the underlying cause [21, 22]. Larger studies are needed to confirm our findings, but proper follow-up for this vulnerable patient cohort must be emphasized. A structured treatment program is necessary after discharge to manage the long-term sequelae of critical illness [9] and should be part of the standard care in all specialized OHCA centers.

A minority of studies reported QoL in the refractory OHCA population [10]. Our data show that a substantial proportion of patients who survived refractory OHCA experience difficulties in daily activities, but the overall QoL assessed by the EQ5D-5L and EQ-VAS show moderate to good QoL in most survivors. Additionally, our results indicate similar QoL in refractory OHCA survivors regardless of the initial treatment strategy. The observational study from Germany also revealed encouraging but impaired QoL in a small group of ECPR recipients [10]. However, a meaningful comparison with our data are not possible as the German study used a different measurement of QoL (i.e., the SF-36 health survey). Our results, with a mean EQ-VAS of 71.0 in the ECPR-based group and 76.3 in the CCPR group, are similar to EQ-VAS results reported in a large TTM2 study (mean EQ-VAS was 74 in the hypothermia group and 75 in the normothermia group) [16].

The limitations of our analysis include those of the primary trial [2]. First, the study was performed in a single high-volume OHCA center experienced in ECMO and ECPR management, restricting the generalizability of our results. Second, the sample size was small, limiting its power. Third, the study design allowed for crossover, which, although occurring at a low rate of 7.5%, may have impacted the results. Fourth, the long-term follow-up has been prospectively conducted but statistical analysis plan was not predefined in the original study protocol, and this is a secondary analysis. Finally, the as-treated and per-protocol analyses should be considered as hypothesis-generating only.

## Conclusions

Among patients with refractory OHCA, the ECPR-based approach significantly improved long-term survival. There were no differences in the neurological outcome, major cardiovascular events and quality of life between the groups but the trial was possibly underpowered to detect a clinically relevant difference in these outcomes. Only a small percentage of long-term survivors experienced severe adverse neurological outcomes. In addition, details from the follow-up reveal many survivors are rehospitalized and encounter difficulties in daily life but their overall QoL is moderate to good. These results highlight the need for comprehensive follow-up for the refractory OHCA population.

### Supplementary Information

**Additional file 1: **Supplementary Tables S1–S5.

## Data Availability

The datasets used and analyzed in this study are available from the corresponding author upon reasonable request.

## Abbreviations

CCPR: Conventional cardiopulmonary resuscitation
CPC: Cerebral performance category
ECMO: Extracorporeal membrane oxygenation
ECPR: Extracorporeal cardiopulmonary resuscitation
mRS: Modified Rankin scale
NYHA: New York Heart Association
OHCA: Out-of-hospital cardiac arrest
QoL: Quality of life
