# Supplementary material for: Effect of intra-arrest transport, extracorporeal cardiopulmonary resuscitation and immediate invasive assessment in refractory out-of-hospital cardiac arrest: a long-term follow-up of the Prague OHCA trial
Source: Crit Care. 2024 Apr 16;28:125. doi: 10.1186/s13054-024-04901-7 (PMC11022382; doi:10.1186/s13054-024-04901-7)
Supplement: Supplementary file 1 — Additional file 1: Supplementary Tables S1–S5. [file 13054_2024_4901_MOESM1_ESM.docx]

**Effect of Intra-arrest Transport, Extracorporeal Cardiopulmonary Resuscitation and Immediate Invasive Assessment in Refractory Out-of-Hospital Cardiac Arrest: A long-term follow-up of the Prague OHCA trial**

**Supplementary Appendix**

**Table of Contents**

**Supplementary Table 1.............................................................................................................3**

**Supplementary Table 2.............................................................................................................5**

**Supplementary Table 3.............................................................................................................6**

**Supplementary Table 4.............................................................................................................7**

**Supplementary Table 5.............................................................................................................8**

**Supplementary Table 1 Neurological outcomes of patients assessed by CPC at 30 days, 180 days, 1 year, 2 years, and at the last follow-up (median 5.3 years, IQR 3.8-7.2 years), by treatment groups, intention-to-treat principle.**

| **CPC at 30-days** | **ECPR-based (n=124)** | **CCPR (n=132)** | **P value*** |
| --- | --- | --- | --- |
| 1 | 27 (21.8 %) | 21 (15.9 %) | 0.02 |
| 2 | 11 (8.9 %) | 3 (22.7 %) |  |
| 3 | 4 (3.2 %) | 7 (5.3 %) |  |
| 4 | 10 (8.1 %) | 12 (9.1 %) |  |
| 5 | 72 (58.1 %) | 89 (67.4 %) |  |
| **CPC at 180-days** |  |  | **P value*** |
| 1 | 34 (27.4 %) | 29 (21.9 %) | 0.09 |
| 2 | 5 (4.0 %) | 0 |  |
| 3 | 0 | 2 (1.5 %) |  |
| 4 | 2 (1.6 %) | 3 (2.3 %) |  |
| 5 | 83 (66.9 %) | 98 (74.2 %) |  |
| **CPC at 1-year** |  |  | **P value*** |
| 1 | 34 (27.4 %) | 29 (21.9 %) | 0.11 |
| 2 | 4 (3.3 %) | 0 |  |
| 3 | 1 (0.8 %) | 2 (1.5 %) |  |
| 4 | 0 | 1 (0.8 %) |  |
| 5 | 85 (68.5 %) | 100 (75.8 %) |  |
| **CPC at 2-years** |  |  | **P value*** |
| 1 | 34 (27.4 %) | 29 (21.9 %) | 0.11 |
| 2 | 4 (3.3 %) | 0 |  |
| 3 | 1 (0.8 %) | 1 (0.8 %) |  |
| 4 | 0 | 1 (0.8 %) |  |
| 5 | 85 (68.5 %) | 101 (76.5 %) |  |
| **CPC at last follow-up (median 5.3 years)** |  |  | **P value*** |
| 1 | 30 (24.4 %) | 25 (18.9 %) | 0.13 |
| 2 | 3 (2.4 %) | 0 |  |
| 3 | 1 (0.8 %) | 1 (0.8 %) |  |
| 4 | 0 | 0 |  |
| 5 | 89 (72.4 %) | 106 (80.3 %) |  |

* The P-value testing was conducted for CPC 1-2 versus CPC 3-5 and mRS 0-3 versus 4-6.

CCPR – Conventional Cardiopulmonary Resuscitation, CPC – Cerebral Performance Category, ECPR – Extracorporeal Cardiopulmonary Resuscitation

**Supplementary Table 2** **Neurological outcome of patients assessed by CPC and mRS at last follow-up, by treatment groups, per protocol analysis.**

* The P-value testing was conducted for CPC 1-2 versus CPC 3-5 and mRS 0-3 versus 4-6.

** The per-protocol analysis is a post-hoc analysis that includes only those patients who completed the treatment originally allocated (excluding all crossovers, 20/256 patients (7.8%) were crossed over, 11 crossovers from the CCPR group to the ECPR-based group and 9 from the ECPR-based group to the CCPR group).

CCPR – Conventional Cardiopulmonary Resuscitation, CPC – Cerebral Performance Category, ECPR – Extracorporeal Cardiopulmonary Resuscitation, mRS – modified Rankin Scale.

| **CPC category** | **Invasive arm (n=114)** | **Standard arm (n=121)** | **P value*** |
| --- | --- | --- | --- |
| 1 | 30 (26.3 %) | 21 (17.4 %) | 0.035 |
| 2 | 3 (2.6 %) | 0 |  |
| 3 | 1 (0.9 %) | 1 (0.8 %) |  |
| 4 | 0 | 0 |  |
| 5 | 80 (70.2 %) | 99 (81.8 %) |  |
| **mRS category** |  |  | **P value*** |
| 0 | 2 (1.8 %) | 5 (4.1 %) | 0.035 |
| 1 | 17 (14.9 %) | 10 (8.3 %) |  |
| 2 | 12 (10.5 %) | 6 (4.9 %) |  |
| 3 | 2 (1.8 %) | 0 |  |
| 4 | 1 (0.9 %) | 0 |  |
| 5 | 0 | 1 (0.8 %) |  |
| 6 | 80 (70.2 %) | 99 (81.8 %) |  |

| **CPC category** | **Invasive arm (n=125)** | **Standard arm (n=130)** | **P value*** |
| --- | --- | --- | --- |
| 1 | 34 (27.2 %) | 21 (16.2 %) | 0.007 |
| 2 | 3 (2.4 %) | 0 |  |
| 3 | 1 (0.8 %) | 1 (0.8 %) |  |
| 4 | 0 | 0 |  |
| 5 | 87 (69.6 %) | 108 (83.1 %) |  |
| **mRS category** |  |  | **P value*** |
| 0 | 3 (2.4 %) | 5 (3.8 %) | 0.007 |
| 1 | 19 (15.2 %) | 10 (7.7 %) |  |
| 2 | 13 (10.4 %) | 6 (4.6 %) |  |
| 3 | 2 (1.6 %) | 0 |  |
| 4 | 1 (0.8 %) | 0 |  |
| 5 | 0 | 1 (0.8 %) |  |
| 6 | 87 (69.6 %) | 108 (83.1 %) |  |

**Supplementary Table 3** **Neurological outcome of patients assessed by CPC and mRS at last follow-up, by treatment groups, as-treated analysis.**

* The P-value testing was conducted for CPC 1-2 versus CPC 3-5 and mRS 0-3 versus 4-6.

** The as-treated analysis is a post-hoc analysis that pooled all randomised patients according to their treatment allocation after the accepted crossover (20/256 patients (7.8%) were crossed over, 11 crossovers from the CCPR group to the ECPR-based group and 9 from the ECPR-based group to the CCPR group).

CCPR – Conventional Cardiopulmonary Resuscitation, CPC – Cerebral Performance Category, ECPR – Extracorporeal Cardiopulmonary Resuscitation, mRS – modified Rankin Scale.

**Supplementary Table 4 Causes of death among patients discharged from the hospital or long-term care hospital facility during the study follow-up, intention-to-treat analysis.**

| **Patient** | **ECPR-based (n=4)** | **CCPR (n=6)** |
| --- | --- | --- |
| 1 | myocardial infarction | sudden cardiac death |
| 2 | lung carcinoma | heart failure |
| 3 | pneumonia | sepsis |
| 4 | urosepsis | heart failure |
| 5 | NA | epilepsy |
| 6 | NA | sudden cardiac death |

CCPR – Conventional Cardiopulmonary Resuscitation, ECPR – Extracorporeal Cardiopulmonary Resuscitation.

**Supplementary Table 5 Detailed causes of rehospitalization during follow-up among patients discharged home from the hospital or long-term facility, intention-to-treat analysis.**

| Hospitalization cause | ECPR-based  (n=30) | CCPR  (n=18) | P-value |
| --- | --- | --- | --- |
| Myocardial infarction | 1 (3.3 %) | 1 (5.6 %) | 0.79 |
| Coronary angiography without revascularization | 6 (20 %) | 2 (11.1 %) | 0.46 |
| Revascularization (PCI/CABG) | 9 (30 %) | 2 (11.1 %) | 0.16 |
| Heart failure | 2 (6.7 %) | 3 (16.7 %) | 0.35 |
| ICD | 5 (16.7 %) | 1 (5.6 %) | 0.29 |
| Aortic valve replacement | 1 (3.3 %) | 1 (5.6 %) | 0.79 |
| Mitral valve replacement | 0 | 1 (5.6 %) | 0.23 |
| Stroke (ischemic) | 0 | 1 (5.6 %) | 0.23 |
| Ventricular tachycardia | 1 (3.3 %) | 3 (16.7 %) | 0.13 |
| Covid-19 pneumonia | 1 (3.3 %) | 1 (5.6 %) | 0.79 |
| Choledocholithiasis with pancreatitis | 1 (3.3 %) | 0 | 0.57 |
| Sepsis | 1 (3.3 %) | 1 (5.6 %) | 0.79 |
| Trauma | 1 (3.3 %) | 0 | 0.57 |
| Bleeding | 1 (3.3 %) | 1 (5.6 %) | 0.79 |

CCPR – Conventional Cardiopulmonary Resuscitation, CABG – Coronary Artery Bypass Grafting, ECPR – Extracorporeal Cardiopulmonary Resuscitation, ICD – Implantable Cardioverter Defibrilator, PCI – Percutaneous Coronary Intervention.
